# Phenotypic Characteristics and Transcriptome of Cucumber Male Flower Development Under Heat Stress

**DOI:** 10.3389/fpls.2021.758976

**Published:** 2021-10-22

**Authors:** Lin Chen, Maomao Yun, Zhenqiang Cao, Zhaojun Liang, Wenrui Liu, Min Wang, Jinqiang Yan, Songguang Yang, Xiaoming He, Biao Jiang, Qingwu Peng, Yu’e Lin

**Affiliations:** ^1^Vegetable Research Institute, Guangdong Academy of Agricultural Sciences, Guangzhou, China; ^2^Guangdong Key Laboratory for New Technology Research of Vegetables, Guangzhou, China

**Keywords:** cucumber, heat stress, pollen fertility, carbohydrate metabolism, RNA-seq

## Abstract

Cucumber (*Cucumis sativus* L.) is an important vegetable crop, which is thermophilic not heat resistant. High-temperature stress always results in sterility at reproductive stage. In the present study, we evaluate the male flower developmental changes under normal (CK) and heat stress (HS) condition. After HS, the activities of peroxidase (POD) and superoxide dismutase (SOD) and the contents of malondialdehyde (MDA) were increased. In addition, the pollen fertility was significantly decreased; and abnormal tapetum and microspore were observed by paraffin section. Transcriptome analysis results presented that total of 5828 differentially expressed genes (DEGs) were identified after HS. Among these DEGs, 20 DEGs were found at four stages, including DNA binding transcription factor, glycosyltransferase, and wound-responsive family protein. The gene ontology term of carbohydrate metabolic process was significantly enriched in all anther stages, and many saccharides and starch synthase-related genes, such as invertase, sucrose synthase, and starch branching enzyme, were significantly different expressed in HS compared with CK. Furthermore, co-expression network analysis showed a module (midnightblue) strongly consistent with HS, and two hub genes (*CsaV3_6G004180* and *CsaV3_5G034860*) were found with a high degree of connectivity to other genes. Our results provide comprehensive understandings on male flower development in cucumber under HS.

## Introduction

In recent years, global warming has resulted in climate changes, including extreme high temperatures, and these changes have caused devastating damage to crop production (Ray et al., 2015). High-temperature stress mainly affects plant seed germination, plant growth, photosynthesis, respiration, water relationship, membrane stability, crop yield, etc. (Kotak et al., 2007; Sehgal et al., 2017; Janni et al., 2020). Nevertheless, plants have developed complex and diverse physiological changes to cope with HS, and many important factors involved in these physiological changes (Alsamir et al., 2021). For example, heat shock proteins, which function as molecular chaperones, will be synthesized in a short time to enhance the heat tolerance of plants and maintain the normal vitality of cells. In addition, antioxidant enzyme systems, including peroxidase (POD), catalase, and superoxide dismutase (SOD), are believed to be necessary for the detoxification of reactive oxygen species (ROS) overproduction under high-temperature stress, which is an important role for maintenance of cell membrane stability (Bita and Gerats, 2013).

In all the processes of plant growth and development, the plant reproductive development stage has the strictest requirements on temperature (Hedhly et al., 2009). The male flower is more sensitive to temperature than female flower (Peet et al., 1998). The damages caused by high temperature to male flowers include pollen abortion, reduce in pollen viability, keep of pollen in anthers and decline pollen germination, degradation of tapetum, formation of pollen wall, destruction of the plasma reticulum in the ribosome, abnormal shape of the vascular bundle cells, the incomplete degradation of the drug barrier, obstruction of the thickening of the inner wall, blocking of the division of anther cells, and so on (Porch and Jahn, 2001; Harsant et al., 2013; Rieu et al., 2017; Shi et al., 2018). In addition, high temperature also changes the carbohydrate metabolism of male flower, for instance, reducing starch accumulation during pollen development, and ultimately leading to pollen abortion (Jain et al., 2010; Cao et al., 2015).

Cucumber, which is one of the most important vegetable crops, is widely cultivated all over the world. The optimum growth temperature of cucumber is between 25 and 30°C, and heat damage will occur if the temperature exceeds 35°C. The cucumbers often encounter high-temperature stress in open-field cultivation or greenhouse cultivation on account of high temperatures in summer in China, which causes the physiological metabolism disorder, the inhibition of growth, abnormal fruit shape and quality, and yield loss (Meng et al., 2004; Sun et al., 2018). A growing number of studies have been reported to heat resistance of cucumber (Li et al., 2016; He et al., 2020; Wang et al., 2020). Ding et al. (2016) reported that exogenous glutathione can enhance cucumber seedling heat tolerance through regulation of antioxidant, osmolytes, and photosynthesis systems. A lot of work has been done on quantitative trait locus (QTL)/gene mapping of heat tolerance in cucumber, such as qHT3.1, qHT3.2, qHT3.3, qHT4.1, qHT4.2, and qHT6.1 (Dong et al., 2020). Liu et al. (2021) identified a heat tolerance QTL named qHT1.1 on chromosome 1 by recombinant inbred lines (RILs). Yu et al. (2018) reported that *CsCaM3* may improve heat tolerance and prevent photosynthetic and oxidative system by regulating the high-temperature responsive genes in cucumber. The male and female flowers of cucumber are most sensitive to high temperature, and the sensitivity of male flower to high temperature is higher than female flower (Miao and Li, 2001). The cucumber pollen showed partial pollen sterility and low pollen vigor when cucumber male flowers are subjected to high temperatures. Furthermore, the meiosis and single microspore stage are the most sensitive to high temperature (Miao, 2000). In addition, the putrescine, spermine, spermidine, and proline content were kept at higher levels in heat resistant varieties compared with heat-sensitive varieties (Miao and Cao, 2002).

Recently, the RNA sequencing (RNA-seq) and microarray technology are powerful and cost-effective tools to investigate the differently crucial genes involved in plant growth and development, abiotic stresses, and biotic stresses (Zhou and Pawlowski, 2014; Fu et al., 2017; Li et al., 2021). The RNA-seq was widely used to understand genes expression involving in abiotic stresses in plant, such as Arabidopsis (Huang et al., 2019), rice (Jin et al., 2013), maize (Guo et al., 2020), tomato (Zhou et al., 2019), wheat (Wang et al., 2019), and eggplant (Zhang et al., 2019). Furthermore, RNA-seq has also been widely applied to investigate the differently expressed genes in cucumber. For instance, transcriptome technique was applied in cucumber to study the floral sex determination (Guo et al., 2010; Wu et al., 2010), fruit curving (Li et al., 2020), fruit spine (Xie et al., 2018), salt stress (Jiang et al., 2020), and disease resistant (Yang et al., 2020). He et al. (2020) reported that cucumber respond to high-temperature stress by full transcriptomic analysis and integrated the potential ceRNA function of lncRNAs/circRNAs for the first time in response to high-temperature stress. However, until now, the differently expressed genes still lack in cucumber male flower after heat stress (HS).

Based on the hypothesis that male flower development is affected in cucumbers under HS, we used physiological and cellular observation and RNA-seq to study the change of male flower. In this study, we thus primarily aimed to evaluate morphological, physiological, and cellular changes in male flower development under normal-temperature and high-temperature stress. It is a comprehensive exploration of the cucumber male flower development under high-temperature stress. In addition, we detected differentially expressed genes (DEGs) in anthers between normal-temperature and high-temperature stress at four development stages by using RNA-seq, which offered insights into the molecular mechanism underlying normal- and high-temperature stress condition. It is a systematic and comprehensive exploration of the cucumber male flower development under high-temperature stress.

## Materials and Methods

### Plant Material and Growth Conditions

A northern type of China cucumber material, B80, which is widely used for breeding in our group, was chosen for this study. Cucumber plants were grown under normal condition until four true leaves. Then, they were transferred to phytotron in normal (CK, 12 h light/12 h dark, 12 h 28°C/12 h 25°C, 70% relative humidity) and HS (12 h light/12 h dark, 12 h 38°C/12 h 30°C, 70% relative humidity) condition, and these states were remained until the end of sampling and investigation. Male flowers were divided into four classes based on male flowers length: 0–3, 3–6, 6–9, and >9 mm, representing S1, S2, S3, and S4, respectively. The anthers were dissected from male flowers at four stages and were collected for three biological replicates.

### Determination of Peroxidase and Superoxide Dismutase Activity and Malondialdehyde Contents in Anther

The anther of CK and HS were collected according to S1, S2, S3, and S4. The activities of POD and SOD and the contents of malondialdehyde (MDA) were measured using kits (Suzhou Keming Bioengineer Company, China). Three biological replicates were performed for all measurements.

### Cytological Observation

The male flowers were harvested between CK and HS, and fixed in Carnoy’s solution (ethanol: acetic acid = 3:1) for 24 h, and they were kept in 70% ethanol after washing three times. The anther was removed from the inflorescences and placed in 1% iodine-potassium (I2-KI), and the normal and abnormal pollens were observed on a microscope (Motic BA200).

The anthers at different development stages were harvested and kept in formaldehyde-acetic acid-ethanol (FAA) (70% ethanol: acetic acid: methanol = 89:6:5), and then embedded in paraffin after dehydration by a series of ethanol concentration according to the protocol of manufacturers. The cross sections of 2 to 5 μm thickness were cut with the microtome (Leica RM2235). The cross sections were stained in 0.05% toluidine blue (m/v) and covered with a slide cover. The sections of anther were observed under a microscope (Motic BA200).

### RNA Sequencing Experiments and Data Analysis

All samples of anther were collected and immediately moved to liquid nitrogen and kept at −80°C for RNA extraction. The RNAs of three biological samples were isolated by TRIzol Reagent (Life technologies, California, United States) according to manual instructions. The libraries were constructed by manual protocols, and transcriptome sequencing was performed on the Illumina Novaseq platform according to the recommended protocol of vendor. Clean reads were obtained by removing reads containing adapter, reads containing N base, and low-quality reads from raw reads. The mapped reads of each sample were assembled by StringTie (v1.3.3b) (Pertea et al., 2015) in a reference-based approach. And then fragments per kilobase of transcript per million fragments mapped reads (FPKM) of each gene were calculated by StringTie. The DEG was detected using the DESeq2 software (Love et al., 2014). The false discovery rate (FDR) was used to determine the threshold of the *P*-value in multiple tests. The DEG was used for subsequent analysis according to the following criteria: FDR ≤ 0.05 and the absolute value of log_2_ (fold change) > 1 or log_2_ (fold change) < −1. The sequencing raw data were also uploaded in National Center for Biotechnology Information (NCBI) (PRJNA748460).

### The Stage-Specific Genes and Weighted Gene Co-expression Network Analysis

The stage-specific genes in anther at different development stages were identified according to the method described previously (Zhan et al., 2015; Garg et al., 2017). The stage specificity coefficient (SSC) score ≥0.5 was used to discover the genes specifically expressed at a special stage.

The weighted gene co-expression network analysis (WGCNA) is a system and comprehensive biology method used to describe gene association patterns between different samples. The co-expression networks analysis was used in WGCNA package in R (Langfelder and Horvath, 2008) according to log2 of the FPKM data of all expressed genes, and analysis is based on the default settings. The kME (eigengene connectivity) values were calculated, and the hub genes in a given module were defined by kME >0.95, which measures the connectivity of a gene in the specific module (Gao et al., 2018).

### Quantitative Real-Time Polymerase Chain Reaction Analysis

The quantitative real-time (qRT)-polymerase chain reaction (PCR) primers were designed by Primer Premier 5.0 software and checked specificity in the NCBI (Supplementary Table 1). The cDNA was from sequenced samples by PrimeScript^TM^ RT reagent Kit with gDNA Eraser (TAKARA), and qRT-PCR was implemented on TB Green Premix Ex Taq^TM^ II (TAKARA). The qRT-PCR reaction condition was similar with Chen et al. (2019), 30 s at 95°C, 40 cycles of 95°C denaturation for 5 s and 60°C annealing. The analysis method was 2^–ΔΔCt^ method to calculate gene-related expression (Livak and Schmittgen, 2001). All qRT-PCR reactions were performed in triplicate.

## Results

### Effects of Heat Stress on Male Reproductive Development

**FIGURE 1:**
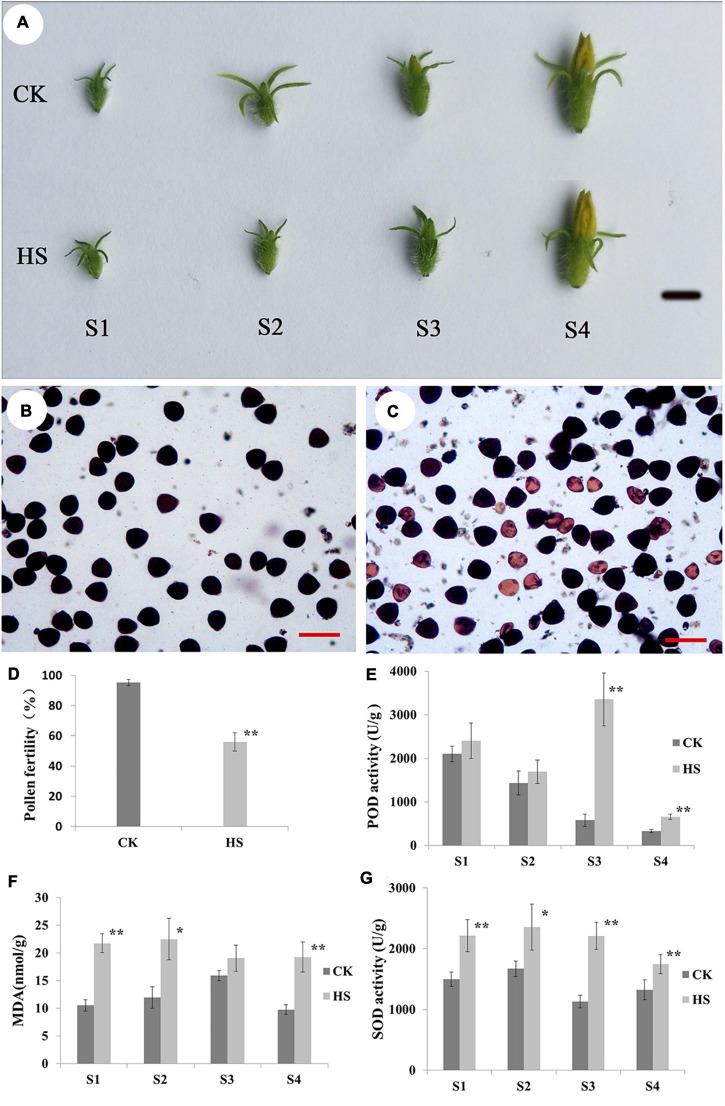
Phenotype of male flower at different developmental stages between normal (CK) and heat stress (HS). **(A)** Phenotype of male flower at different developmental stages between CK and HS, bar = 1cm. Pollen grain of CK **(B)** and HS **(C)** observed by staining with 1% iodine-potassium (I_2_-KI), pollen fertility **(D)**, bar = 100 μm. Peroxidase (POD) **(E)**, malondialdehyde (MDA) **(F)**, and superoxide dismutase (SOD) **(G)** in anther of CK and HS at male flower development stages.

To study the cucumber responses to HS at reproduction stage, the cucumber plants were moved to grow under CK (12 h light/12 h dark, 12 h 28°C/12 h 25°C) and HS (12 h light/12 h dark, 12 h 38°C/12 h 30°C) when seedlings were with four true leaves. There are no differences on male flower between HS and CK condition (Figure 1A). To investigate how pollen fertility was affected by HS, pollen viability was tested using I_2_-IK staining. As shown in Figures 1B–D, many mature pollen grains were aborted on HS, and pollen fertility (55.92%) was significantly lower than CK. In addition, we investigated SOD enzyme activity, POD activity, and the contents of MDA at S1, S2, S3, and S4 (see detail in materials and methods section) (Figures 1E–G). In POD activity, there were extremely significant increase at S3 and S4 between HS and CK (Figure 1E). The MDA contents of HS were higher than CK, and there were significant or extremely significant differences between HS and CK at S1, S2, and S4 (Figure 1F). The results of SOD activity were significant or extremely significant differences at every anther development stage between HS and CK (Figure 1G).

**FIGURE 2:**
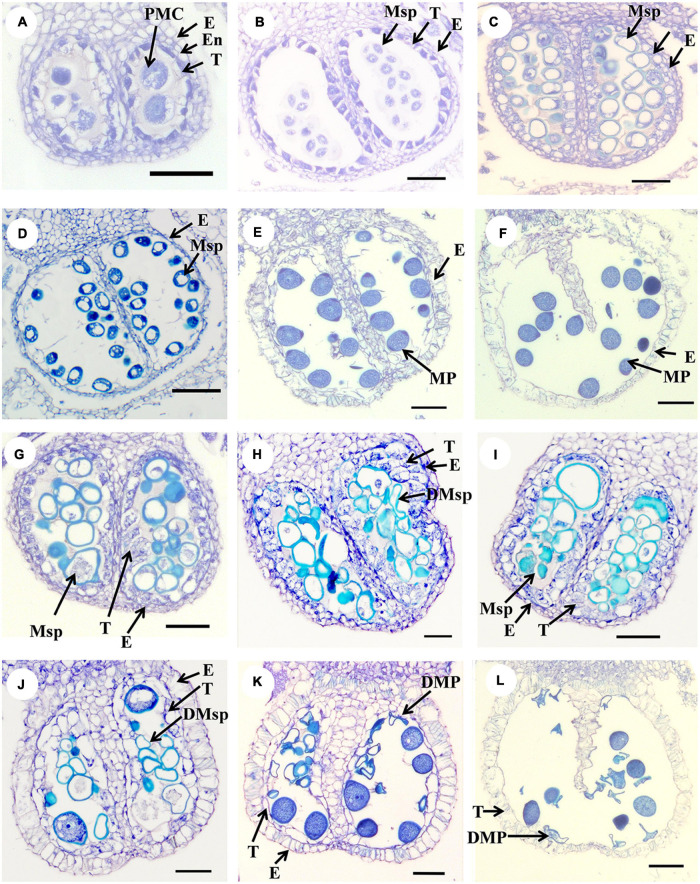
The cross-section analysis of anther development in CK and HS. **(A–F)** Anther cross-sections of different developmental stages under CK, **(A–B)** meiosis stage, **(C–D)** microspore stage, **(E–F)** and mature stage. **(G–L)** Abnormal anther in HS. bar = 50 μm.

The cross section assay was carried out to investigate anther development in CK and HS by paraffin section (Figure 2). Many abnormal phenomena were found since microspore stage to mature pollen stage underlying HS. At microspore stage, there were tapetum abnormality, abnormal microspore, and microspore degeneration in HS (Figures 2G–I). At mature pollen stage, the pollen grains were filled with starch and tapetum wasdegenerated in CK. However, the pollen grains became sterile and tapetum did not degenerate in HS (Figures 2J–L). Taken together, these results showed that HS has a large impact on anther development.

### Transcriptomic Profiles of Cucumber Anther Under Heat Stress

About 102 million clean reads were obtained after moving low quality read in different samples. We mapped the clean reads to cucumber reference genome (V3.0), and 91.07% to 94.52% clean reads were uniquely mapped to the reference genome in different samples (Supplementary Table 2). The correlation coefficient of each sample was more than 0.9 (Supplementary Figure 1), and principal component analysis (PCA) showed that biological replicates were clustered together (Supplementary Figure 1) and sample of each stage was classed together. These results indicated that gene expression patterns have high similarity between biological replications and sample of each stage have similar expression pattern.

### Specifically Different Expression at Anther Development

**FIGURE 3:**
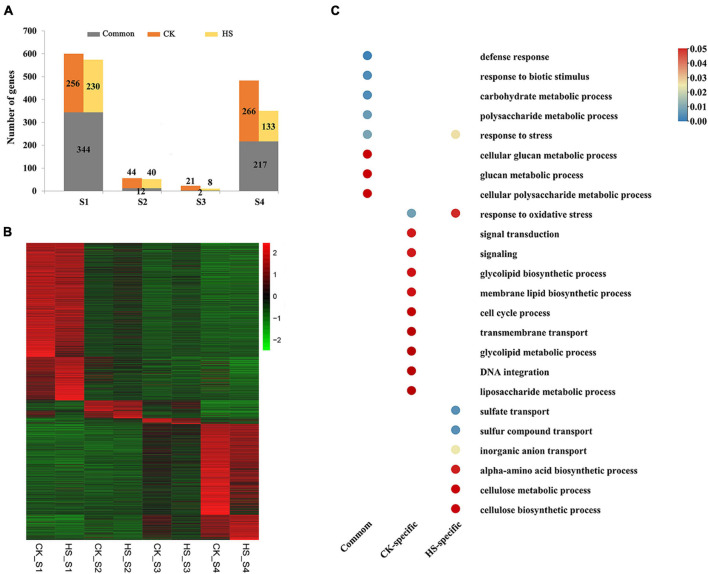
Specifically expressed genes at male development stages in cucumber. **(A)** The number of specifically expressed genes specifically and commonly in CK and HS at each stage of anther development. **(B)** The expression profile of specifically expressed genes between in CK and HS. Color scale represents Z-score. **(C)** The significantly enriched gene ontology (GO) terms (biological process) of specifically expressed genes at anther development in CK and HS.

To investigate the specifically different expression at different anther development stages under different growth conditions, we identified specifically expressed genes in different anther development stages by using SSC. Total of 1162 and 986 specifically expressed genes were discovered at a particular sample on normal and HS condition, respectively (Figure 3A). The stage-specific genes varied from 23 to 600 for CK and 10 to 574 for HS (Figure 3A). The S1 presented the largest number of stage-specific genes and the lowest number of stage-specific genes was detected in S3 in both growth conditions (Figures 3A,B). More stage-specific genes were found in S1 and S4 stages; this indicated these two stages are more active.

The gene ontology (GO) enrichment analyses showed that all the stage-specific genes were related to various transport, response, biosynthetic, and metabolic processes (Supplementary Table 2). These GO terms are well known to be related to the growth and development of plants. Total of 55, 87, and 51 GO terms were significantly enriched in CK, HS, and both growth conditions by enrichment stage-specific genes, respectively (Figure 3C and Supplementary Figure 2). In the biological processes, GO terms of response to oxidative stress and response to stress were significantly enriched in both growth conditions, and other GO terms are specifically significantly enriched in one growth condition. For example, signal transduction, glycolipid biosynthetic process, membrane lipid biosynthetic process, cell cycle process, transmembrane transport, glycolipid metabolic process, DNA integration, and liposaccharide metabolic process were specific to CK, whereas GO terms related to sulfate transport, sulfur compound transport, inorganic anion transport, alpha-amino acid biosynthetic process, cellulose metabolic process, and cellulose biosynthetic process were specific to HS. The GO terms, including defense response, response to biotic stimulus, carbohydrate metabolic process, polysaccharide metabolic process, cellular glucan metabolic process, glucan metabolic process, and cellular polysaccharide metabolic process, were identified enrichment in common stage-specific genes between both growth conditions (Figure 3C). These results indicate that a group of genes implement stage-specific functions during anther development stage between both growth conditions.

### Differentially Expressed Genes in Male Reproductive Under Heat Stress

**FIGURE 4:**
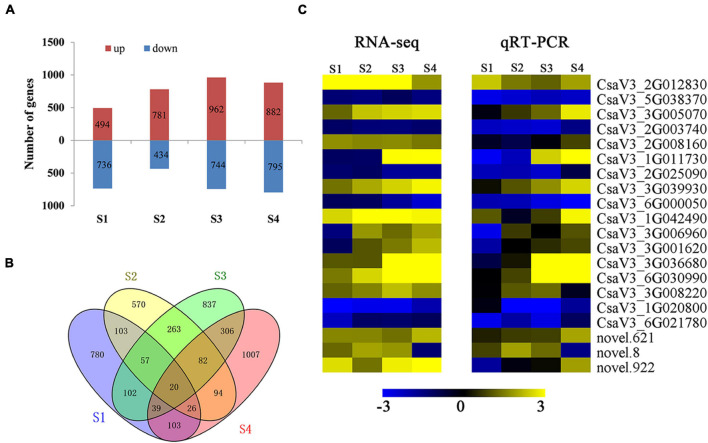
Differentially expressed genes (DEGs) in HS compared with CK at anther development stages. **(A)** Number of up and downregulated DEGs in a HS compared with CK. **(B)** Venn diagram of DEGs in HS compared with CK. **(C)** Expression patterns of common DEGs by RNA-seq and qRT-PCR.

We detected DEGs with heat-induced expression change at anther development stage. We identified 1230, 1215, 1706, and 1677 DEGs in S1, S2, S3, and S4 in HS compared with CK (Figure 4A), respectively. Additionally, 20 DEGs were significantly expressed at every development stage in HS compared with CK (Figure 4B). These 20 DEGs were also selected for qRT-PCR analysis and confirmed this prediction outcome (Figure 4C). Totally, six and 10 DEGs were detected significantly downregulated and upregulated at all stages in HS compared with CK, and four DEGs differently significantly expressed in different stage. The six DEGs were detected significantly downregulated in HS, including chorismate mutase (*CsaV3_5G038370*), threonine aldolase (*CsaV3_2G00374*0), pectate lyase (*CsaV3_2G025090*), hexosyltransferase (*CsaV3_ 6G000050*), xyloglucan endotransglucosylase (*CsaV3_1G020 800*), and syntaxin (*CsaV3_6G021780*). The 10 DEGs were detected significantly upregulated in HS, including Reactive intermediate/imine deaminase (*CsaV3_3G005070*), (R)-mandelonitrile lyase (*CsaV3_2G008160*), protein NRT1 (*CsaV3_ 3G039930*), Beta-glucosidase (*CsaV3_1G042490*), sequence-specific DNA binding transcription factor (*CsaV3_3G036680*), fatty acid desaturase (*CsaV3_6G030990*), Glycosyltransferase (*CsaV3_3G008220*), and three unknown protein (*CsaV3_ 2G012830*, *novel.621*, and *novel.922*). The four DEGs differently expressed in different stages, including wound-responsive family protein (*CsaV3_1G011730*), dof zinc finger protein like (*CsaV3_3G006960*), glycine-rich protein-2 (*CsaV3_3G001620*), and unknown protein (*novel.8*). These genes may have key roles in anther development and pollen formation, and may be used as marker gene to distinguish HS and CK conditions.

Total of 416 DEGs belong to 58 transcription factors (TFs), and including 93, 119, 121, and 84 TFs were found in S1, S2, S3, and S4 in HS compared with CK (Supplementary Figure 3A), respectively. The TFs of MYB, ERF, ERF, and bHLH were mostly detected in S1, S2, S3, and S4 in HS compared with CK (Supplementary Figure 3B), respectively. We further analyzed heat shock transcription factors (HSFs) in different development stages and detected six HSFs (three upregulated and three downregulated) in S2, five HSFs (one upregulated and four downregulated) in S3, and one downregulated HSF in S4 (Supplementary Table 4). In particular, *CsaV3_2G002270* was downregulated in S2 and S3, and *CsaV3_7G027860* was downregulated in S2, S3, and S4. *CsaV3_3G036820* was upregulated in S2 and S3. In addition, 18 heat shock proteins (HSPs) were found significantly different expressed in different development stages, and 11 HSPs (four upregulated and seven downregulated) in S1, four HSPs (three upregulated and one downregulated) in S3, six HSPs (five upregulated and one downregulated) in S3, and two downregulated HSPs in S4 (Supplementary Table 4). Interestingly, the *CsaV3_1G035830* was downregulated in S1, S3, and S4.

**FIGURE 5:**
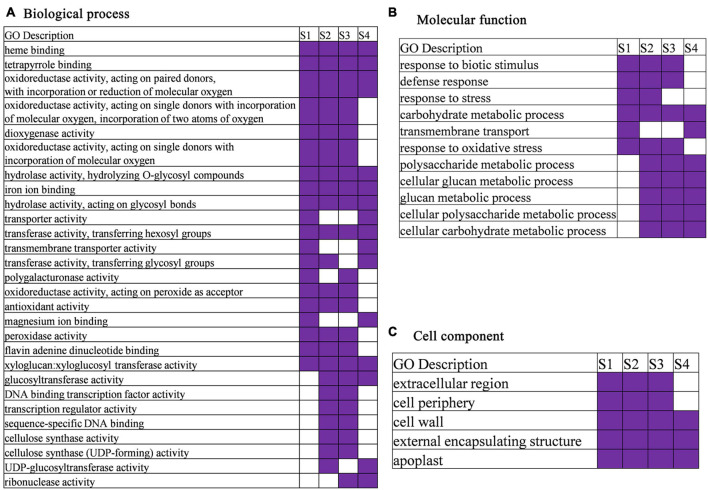
Gene ontology (GO) enrichment heatmap for DEGs in anther development stage (GO terms were selected based on their appearance at least in two times or more). **(A)** Biological process. **(B)** Molecular function. **(C)** Cell component.

The GO analysis presented that 42, 68, 51, and 80 GO terms were significantly enriched in S1, S2, S3, and S4, respectively (Figure 5 and Supplementary Table 4). In the biological processes category, carbohydrate metabolic process was enriched at every stage. The GO terms of response to biotic stimulus, defense response, and response to oxidative stress were significantly enriched in S1, S2, and S3, and response to stress (response to biotic stimulus, defense response, and response to oxidative stress are subterm of response to stress) was significantly enriched in S1 and S2. A total of eight GO terms, such as heme binding, tetrapyrrole binding, iron ion binding, hydrolase activity, and acting on glycosyl bonds, were identified in molecular function category at every development stage. In cell component category, GO terms related to cell wall, external encapsulating structure, and apoplast at every development stage were identified (Figure 5 and Supplementary Table 4).

### Differently Expressed Genes Involved in Carbohydrate Metabolism

**FIGURE 6:**
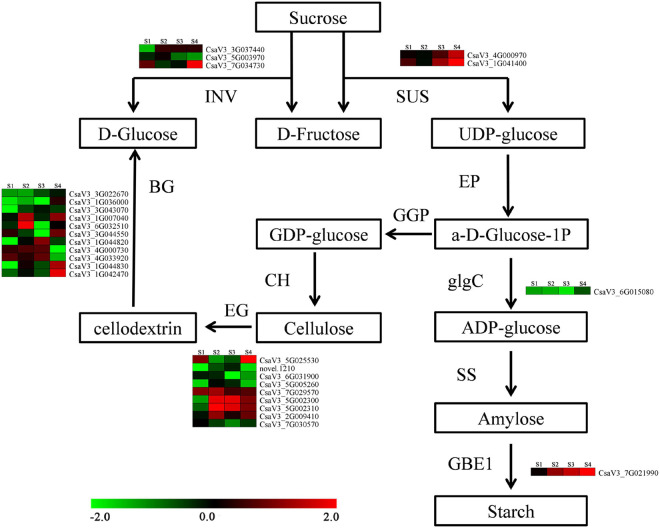
Predicted carbohydrate metabolism pathways in different anther development under HS. INV: invertase; SUS: sucrose synthase; EP: ectonucleotide pyrophosphatase; glgC: glucose-1-phosphate adenylyltransferase; SS: starch synthase; GBE1: glucan branching enzyme; CH: cellulose synthase; EG: endoglucanase; BG: beta-glucosidase.

A total of 121 DEGs involved in carbohydrate metabolic process were differentially expressed on anther at four stages between CK and HS, including 75 DEGs are encode glycosyl hydrolase protein, and three DEGs are translated glycosyl transferase protein. There are 58 DEGs that were downregulated in HS, and 55 DEGs were upregulated in HS, and eight DEGs were differently expressed pattern between HS and CK. *CsaV3_1G036000*, *CsaV3_5G005550*, and *CsaV3_1G020800* were downregulated at S1, S2, and S3. *CsaV3_3G036070* was upregulated at S1, S3, and S4, and *CsaV3_7G021990*, *CsaV3_1G031460*, and *CsaV3_2G016560* were upregulated at S2, S3, and S4. In particular, *CsaV3_1G042490* was upregulated at all stages (Supplementary Figure 4). In addition, there are many DEGs that were involved in starch and sucrose metabolism and sucrose transporters (Figure 6). The three sucrose transporter genes (*CsaV3_2G010710*, *CsaV3_2G010690*, and *CsaV3_2G010720*) were detected as significantly different expressed HS compared with CK. The invertase (INV) (*CsaV3_3G037440*, *CsaV3_5G003970*, and *CsaV3_7G034730*), sucrose synthase (SUS) (*CsaV3_4G000970* and *CsaV3_1G041400*), and starch branching enzyme (*CsaV3_7G021990*) displayed different expression patterns between HS and CK (Figure 6).

### Co-expression Network Analyses Different Anther Development Stage by Weighted Gene Co-expression Network Analysis

The WGCNA was constructed to understand the gene regulatory network in response to HS on anther at different development stages. A total of 12781 genes were used for WGCNA, and distributed to 17 modules (Supplementary Figures 5A,B and Supplementary Table 6). The number of genes in these modules varied from 15 (MEgrey) to 4931 (MEturquoise), and the modules harbored TF genes varying 1 (MElightblue) to 319 (MEblue) (Supplementary Figure 5C).

### Identification of Heat Response-Related Modules

**FIGURE 7:**
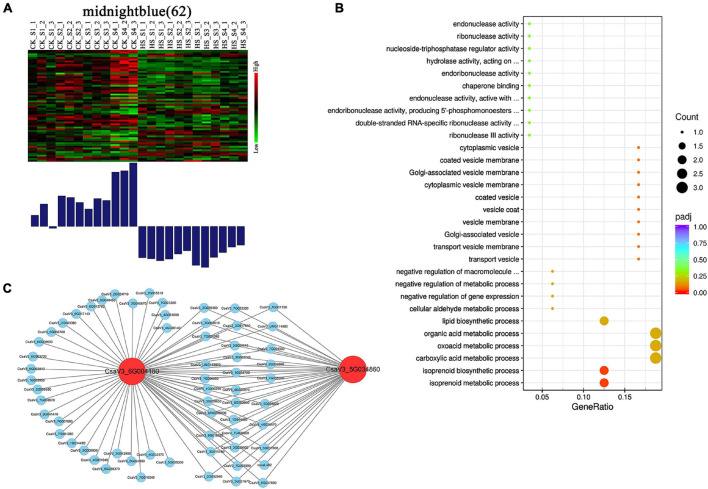
Expression profile and transcriptional regulatory network of midnightblue module associated with opposite expression patterns in CK and HS. **(A)** The midnightblue modules showing opposite expression patterns in CK and HS. Heatmaps show the expression profile of all the co-expressed genes (number given on the top) in the midnightblue modules. The color scale represents Z-score. Bar graphs (below the heatmaps) show the consensus expression pattern of the co-expressed genes in midnightblue modules. The bars show opposite expression patterns in CK and HS. **(B)** The GO terms (biological process) of genes in midnightblue modules. **(C)** High co-expressed genes of two hub genes in midnightblue module.

We found that some modules showed different expression patterns between CK and HS at different development stages. For example, the tan module showed low expression in CK, but high expression in HS at S3 and S4 stages. The pink module showed low expression in CK, but high expression in HS at S1, S2, and S3 stages. Likewise, the black module showed low expression in HS, but high in CK at S3 stage. The red module showed low expression in HS, but high expression in CK at S4 stage. The green and lightcyan module showed low expression in HS, but high expression in CK at S4 stage. The magenta module showed low expression in HS, but high expression in CK at S2 and S3 stages (Supplementary Figure 6). Particularly, the midnightblue module was preferential expression in CK, and lower expression in HS at all anther development stages (Figure 7A).

The midnightblue modules, including 62 genes, may be related to HS in anther development, and we further analyzed the genes of midnightblue modules. The GO enrichment analysis revealed that the midnightblue module was enriched for many metabolic processes in biological processes, including isoprenoid metabolic process, carboxylic acid metabolic process, and organic acid metabolic process (Figure 7B). The hub genes, which can be deemed as representative of the gene expression profile of the module, were identified by kME value. The genes (kME > 0.95) were chosen as hub genes in each module. Two genes (*CsaV3_6G004180* and *CsaV3_5G034860*) were found as hub genes in midnightblue module (Supplementary Table 7). Functional annotations showed that *CsaV3_6G004180* encodes ACT (aspartokinase, chorismate mutase and TyrA) domain containing protein and *CsaV3_5G034860* might be repressor of RNA polymerase III transcription. We visualized genes with high correlation to *CsaV3_6G004180* and *CsaV3_5G034860* (Figure 7C). Notably, *CsaV3_6G004180* and *CsaV3_5G034860* shared 33 overlapped correlation genes, such as WRKY factor (*CsaV3_3G004410*) and zinc finger protein (*CsaV3_4G030470*) and calcium-dependent protein kinase (*CsaV3_3G017510)*.

## Discussions

### High Temperature Effects Pollen Fertility

Plants are sensitive to high temperature at reproductive development, which can cause male or female sterility (Hedhly et al., 2009). Furthermore, many studies found that male reproductive organs were more susceptible to HS than female reproductive organs (Peet et al., 1998). In cucumber, high temperature changed many physiological characteristics, including MDA content, catalase, glutathione reductase, guaiacol peroxidase (G-POD), and SOD activities (Ding et al., 2016). In the current study, we explored phenotype and physiology in cucumber anther development under HS. The results showed that the anthers are no obviously different feature, but the SOD, POD, and MDA present difference in anther between CK and HS. The physiological investigation results are consisted with other studies on high-temperature stress. In rice, the pollen fertility markedly reduced when rice experienced high temperature at pollen development stage, including reduced number of pollen grain, decreased pollen vitality, and abnormal pollen morphology (Endo et al., 2009; Shi et al., 2013; Kumar et al., 2019). Moreover, high temperatures also cause pollen sterility in cucumber (Miao, 2000). In this study, cytological results presented many abnormal anthers were found in HS, including tapetum swollen, tapetum delayed degradation, degraded microspore, and aborted pollen. Furthermore, the pollen fertility was significantly reduced in HS compared with CK. These results indicated that high temperature may harm the development of cucumber male flowers and causes partial sterility of pollen.

### The Abrupt Expression of Saccharides and Starch Synthase-Related Genes May Cause Partial Pollen Sterility by Heat Stress

It is well known that carbohydrate metabolism is essential for plant growth and development. As the main nutrients, the carbohydrates supply energy for the development of pollen (Ruan et al., 2010). In addition, Chen et al. (2018) reported that abnormal saccharides distribution pattern and abrupt saccharides-related genes expression may decrease pollen fertility in autotetraploid rice. Moreover, the starch-related genes were mis-regulation in mature pollen after heat treatment in maize (Begcy et al., 2019), and many proteins of starch and sucrose metabolism were increased expression in pollen under heat treatment in cotton (Masoomi-Aladizgeh et al., 2021). Here, the genes expression profile analysis suggested that carbohydrate metabolic process was detected significantly enriched in DEG at each stage between CK and HS (Figure 5). The SUS and INV are two important enzymes involving sucrose degradation in plants (Ruan, 2012). Here, three SUSs and two INVs were identified to significantly express in HS and CK. There are three enzymes that catalyze the starch synthesis and metabolism in plants, including starch synthase, starch debranching, and starch branching enzyme (Zeeman et al., 2010). The starch branching enzyme (*CsaV3_7G021990*) genes was upregulated at S2, S3, and S4 in HS compared with CK. The endoglucanase is necessary for normal cellulose synthesis and has effects on plant cell growth (Read and Bacic, 2002). In our results, nine endoglucanase genes were detected significantly different expressed between HS and CK. The β-glucosidase is extensively involved biotic and abiotic stresses in plant (Xu et al., 2012; Zamioudis et al., 2014), and β-glucosidases gene takes part in the development of pollen in *Brassica species* (Dong et al., 2019). A total of 11 DEGs, which encode β-glucosidases, were found between HS and CK.

Sucrose transporters support translocation of sucrose from source to sink organs, and maintain connection between sucrose transport and pollen development (Srivastava et al., 2008; Hirose et al., 2010). *CsSUT1*, which is a sucrose transporter protein, was found to be important for male sterility (Sun et al., 2019). The *CsSUT1* was found upregulated at S4 in HS, and two sucrose transporters (*CsaV3_2G010710* and *CsaV3_2G010720*) were detected. These results showed that many saccharides metabolism, starch synthase, and sucrose transporter-related genes were significantly expressed in HS and CK, and consisted with maize (Begcy et al., 2019) and cotton (Masoomi-Aladizgeh et al., 2021). We conjectured that those abrupt expressions of saccharides metabolism and starch synthase-related genes may cause low pollen fertility in HS.

### Heat Shock Protein and Heat Shock Transcription Factor Play an Important Role in Anther Responding to High Temperature

It has been reported that HSP and HSF play a crucial role for responding to HS. HSF is a transcription factor necessary to induce HS response, and HSFs are evolutionarily conserved in eukaryotes (Ohama et al., 2017). According to the characteristics of their flexible linkers and oligomerization domains, the HSFs are divided into three categories (A, B, and C) (Nover et al., 2001; Bharti et al., 2004). In Arabidopsis, *HsfA3* was high expression at later phase of the high-temperature response, and it is an important factor in thermotolerance network by forming complexes with other HSFs (Schramm et al., 2008). In current study, eight HSFs were significantly different expressed in CK and HS, including six, six, and one HSFs were detected at S2, S3, and S4, respectively. In particular, a HSF (*CsaV3_7G027860*) was significantly downregulated in HS at S2, S3, and S4. In addition, the HSP, such as HSP60, HSP70, HSP90, and HSP21, respond broadly to HS by regulating protein quality (Qu et al., 2013). In Arabidopsis, *HSP21* may regulate plastid encoded RNA polymerase transcription to maintain the normal development of chloroplasts under high-temperature stress (Zhong et al., 2013). The HSP appeared differently expressed in cotton pollen under high-temperature stress (Masoomi-Aladizgeh et al., 2021). Here, 18 HSPs were significantly different expressed in CK and HS, including 11, four, six, and two HSPs were detected at S1, S2, S3, and S4, respectively. There was a HSP (*CsaV3_1G035830*), which was significantly downregulated in HS at S1, S3, and S4. These results showed these HSFs and HSPs have essential roles under HS and may be important factors for cucumber anthers responding to HS at male flowering development stages.

### Transcription Regulatory Modules Related to Heat Stress in Cucumber Anther Development

Weighted gene co-expression network analysis (WGCNA), which is a systems analysis tool, was used to identify large gene clusters and relationships of networks and phenotypes. The WGCNA is widely used in rice, tomatoes, chickpea, strawberry, and soybean. In the present study, WGCNA were used to detect that gene different expressed pattern between HS and CK. A module (midnightblue) was identified as oppositely expressed pattern between HS and CK at all anther development stages. In addition, WGCNA analysis found two hub (*CsaV3_6G004180* and *CsaV3_5G034860*) genes in midnightblue module (Figure 7). *CsaV3_6G004180* is ACT domain containing protein. ACT domain containing protein can interact with small heat stress protein (Hayakawa et al., 2006), and may regulate ROS and salicylic acid (SA) accumulation to modulate SA-associated defense responses and disease resistance in Arabidopsis (Singh et al., 2018). *CsaV3_5G034860* encoded repressor of RNA polymerase III transcription. The RNA polymerase III was essential for reproductive development (Onodera et al., 2008), and loss of RNA polymerase III will cause hybrid pollen sterility in rice (Nguyen et al., 2017). On further analysis, 33 genes were highly correlated with *CsaV3_6G004180* and *CsaV3_5G034860*. The *CsaV3_6G004180*, and *CsaV3_5G034860* play an important related role in adjusting HS in anther development.

Although it is well known that cucumber is sensitive to HS at male flower development stage, the molecular mechanisms are poorly understood in cucumber male flower development underlying HS. In this study, we analyzed physiological, cellular changes, and transcriptomes data in male flower between CK and HS. The results showed that physiological levels, including POD, SOD, and MDA, are affected by HS. The many HSPs and HSFs were observed to be abrupt and could affect flower development as well in HS. Moreover, the carbon utilization genes were found to change and might be the cause of partial pollen sterility in HS. These results revealed that multiple reasons jointly cause partial pollen abortion in cucumber under HS.

## Data Availability Statement

The datasets presented in this study can be found in online repositories. The names of the repository/repositories and accession number(s) can be found below: NCBI SRA BioProject, accession no: PRJNA748460.

## Author Contributions

LC and YL designed the experiment. LC, MY, ZC, ZL, WL, MW, JY, XH, BJ, and QP performed most of the experiments. LC wrote the manuscript. SY edited the manuscript. All authors read and approved the final version of the manuscript.

## Conflict of Interest

The authors declare that the research was conducted in the absence of any commercial or financial relationships that could be construed as a potential conflict of interest.

## Publisher’s Note

All claims expressed in this article are solely those of the authors and do not necessarily represent those of their affiliated organizations, or those of the publisher, the editors and the reviewers. Any product that may be evaluated in this article, or claim that may be made by its manufacturer, is not guaranteed or endorsed by the publisher.
