# Supplementary material for: Phenotypic Characteristics and Transcriptome of Cucumber Male Flower Development Under Heat Stress
Source: Front Plant Sci. 2021 Oct 22;12:758976. doi: 10.3389/fpls.2021.758976 (PMC8570340; doi:10.3389/fpls.2021.758976)
Supplement: Supplementary file 1 [file Data_Sheet_1.zip › Supplement information/Supplement Figure .pdf]

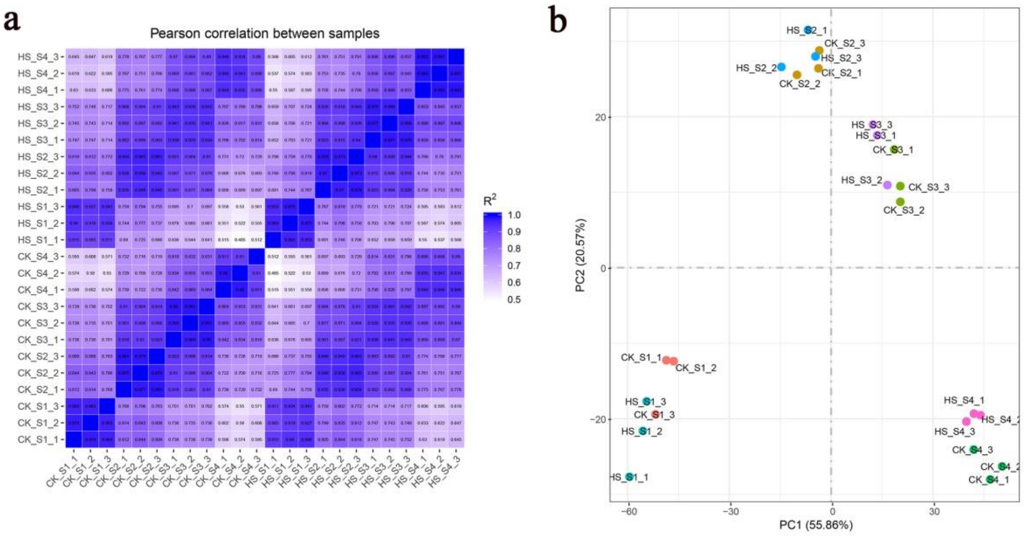

Figure S1 The correlation coefficient (a) and principal component analysis (b).

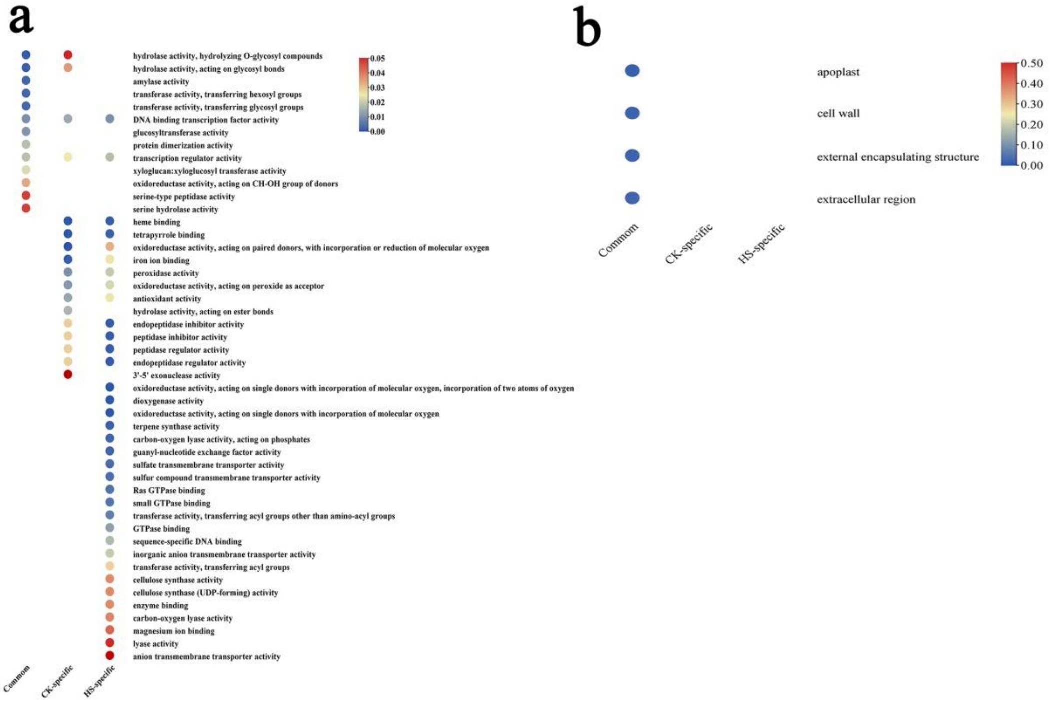

Figure S2 The significantly enriched GO terms of specifically expressed genes at anther development in CK and HS. a, molecular function, b, cell component.

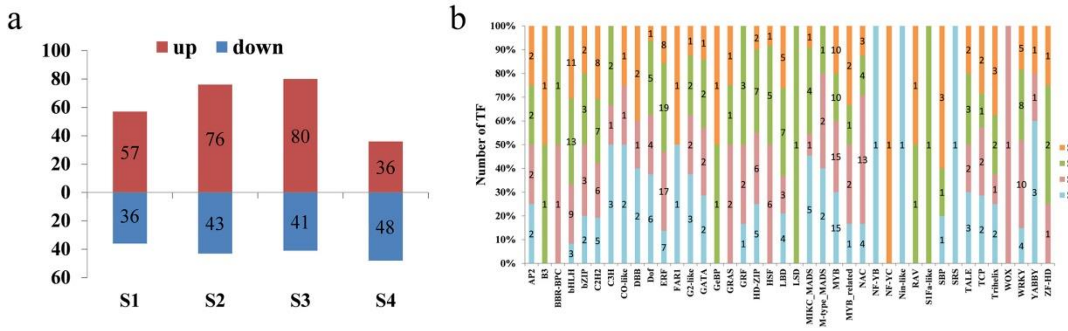

Figure S3 The number of transcription factors (TFs) up- or down-regulated and the number of DEGs belonging to different transcription factor families.

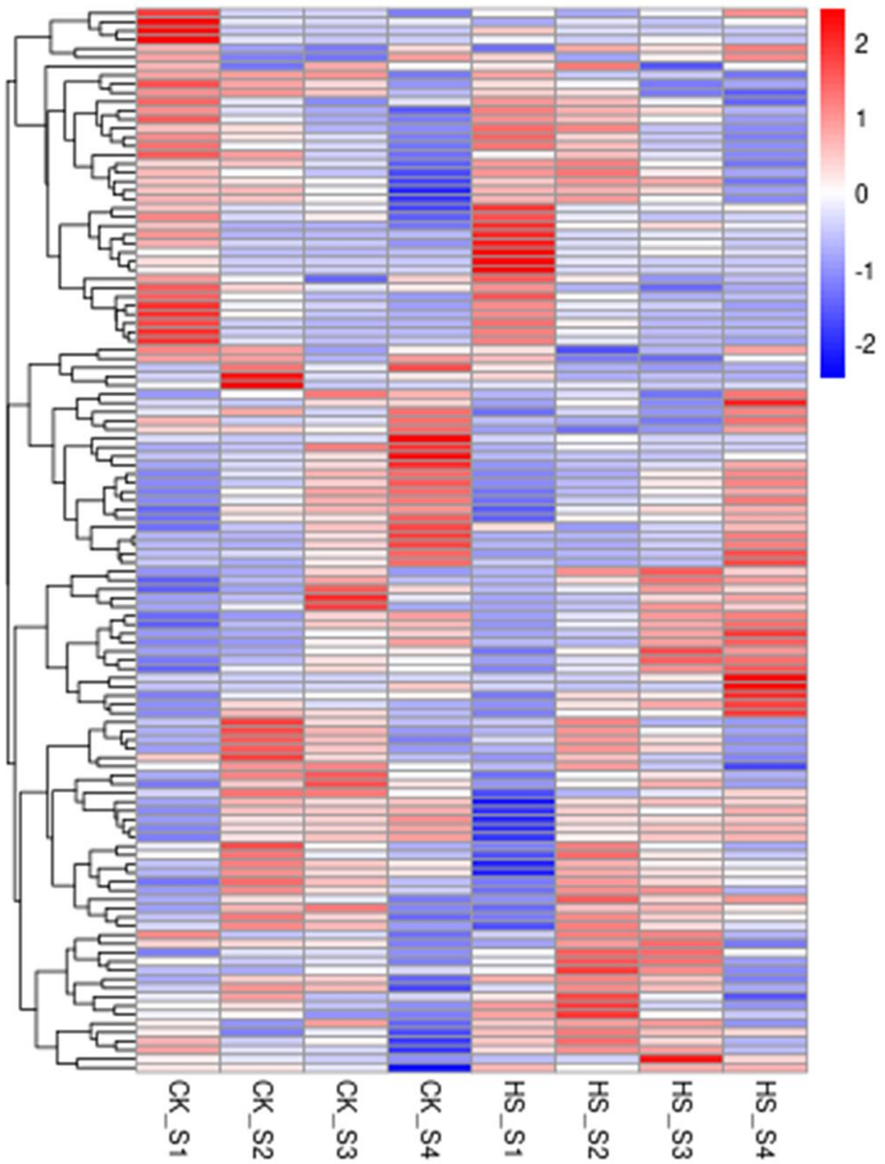

Figure S4 Heatmaps of DEGs encoding proteins involved in carbohydrate metabolism.

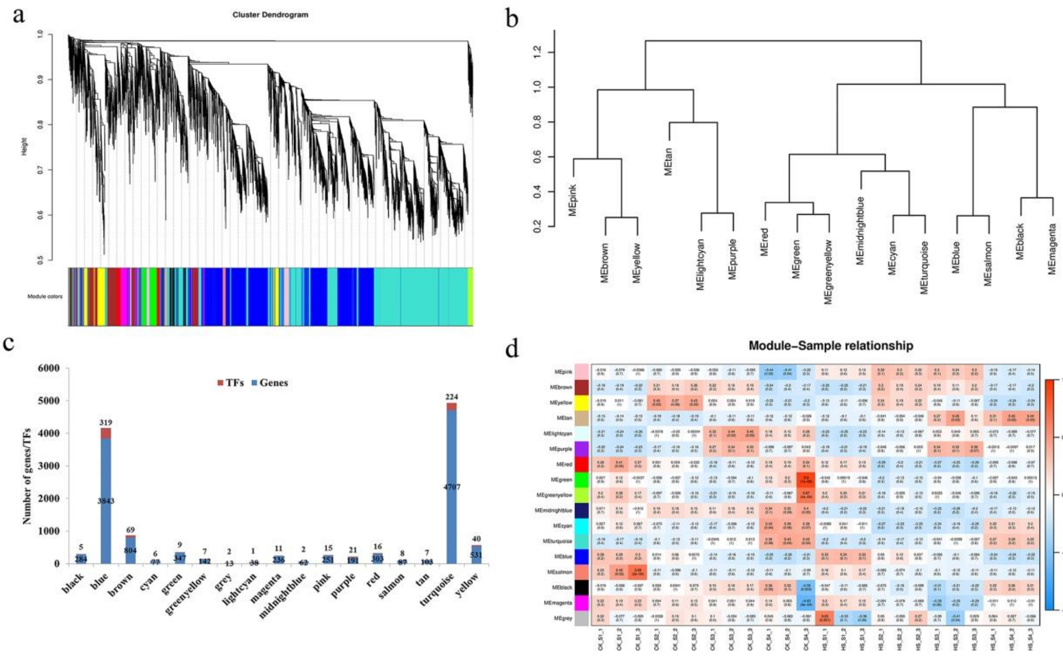

Figure S5 WGCNA based gene expression matrix between CK and HS.

a. Hierarchical cluster tree showing co-expression modules identified by WGCNA, b. Dendrogram of module eigengenes, c. Number of gene and transcription factors (TFs) in different module, d. Module-sample relationship.

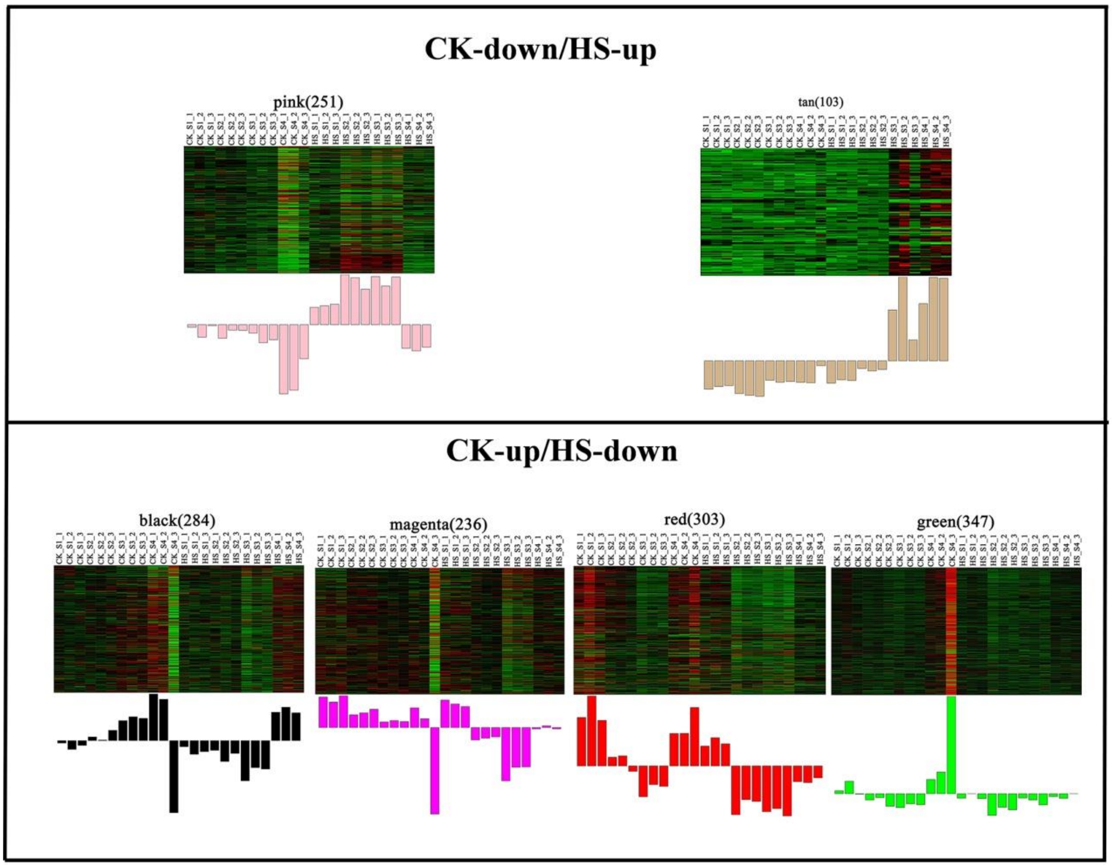

Figure S6 Expression profile of the modules associated with CK and HS.
